# Rumen Microbiota Distribution Analyzed by High-Throughput Sequencing After Oral Doxycycline Administration in Beef Cattle

**DOI:** 10.3389/fvets.2020.00251

**Published:** 2020-06-02

**Authors:** Fengmei Chen, Guangmin Cheng, Yulin Xu, Yunzhou Wang, Qingxiang Xia, Shilin Hu

**Affiliations:** ^1^Shandong Research Center for Technology of Reduction of Antibiotics Administered to Animal and Poultry, Shandong Vocational Animal Science and Veterinary College, Weifang, China; ^2^Comparative Medicine Research Institute, Yangzhou University, Yangzhou, China; ^3^College Veterinary Medicine, Yangzhou University, Yangzhou, China; ^4^Jiangsu Co-innovation Center for Prevention and Control of Important Animal Infectious Diseases and Zoonoses, Yangzhou, China; ^5^Joint International Research Laboratory of Agriculture and Agri-Product Safety, Yangzhou University, Yangzhou, China

**Keywords:** beef cattle, rumen microbiota, doxycycline, MiSeq sequencing, dysbacteriosis, oral antibiotics, bacterial richness

## Abstract

The beef cattle rumen is a heterogenous microbial ecosystem that is necessary for the host to digest food and support growth. The importance of the rumen microbiota (RM) is also widely recognized for its critical roles in metabolism and immunity. The level of health is indicated by a dynamic RM distribution. We performed high-throughput sequencing of the bacterial 16S rRNA gene to compare microbial populations between rumens in beef cattle with or without doxycycline treatment to assess dynamic microbiotic shifts following antibiotic administration. The results of the operational taxonomic unit analysis and alpha and beta diversity calculations showed that doxycycline-treated beef cattle had lower species richness and bacterial diversity than those without doxycycline. Bacteroidetes was the predominant phylum in rumen samples without doxycycline, while Proteobacteria was the governing phylum in the presence of doxycycline. On the family level, the top three predominant populations in group qlqlwy (not treated with doxycycline) were Prevotellaceae, Lachnospiraceae, and Ruminococcaceae, compared to Xanthomonadaceae, Prevotellaceae, and Rikenellaceae in group qlhlwy (treated with doxycycline). At the genus level, the top predominant population in group qlqlwy was unidentified_Prevotellaceae. However, in group qlhlwy, the top predominant population was Stenotrophomonas. The results revealed significant RM differences in beef cattle with or without doxycycline. Oral doxycycline may induce RM composition differences, and bacterial richness may also influence corresponding changes that could guide antibiotic use in adult ruminants. This study is the first to assess microbiota distribution in beef cattle rumen after doxycycline administration.

## Highlights

- This is the first study of cattle rumen microbiota after doxycycline treatment.
- Doxycycline-treated beef cattle have lower species richness and diversity.
- Bacteroidetes was the most dominant phylum in untreated rumen samples.
- Proteobacteria was the governing phylum in the presence of doxycycline.

## Introduction

Ruminants convert human-inedible plant biomass into meat and dairy products with high nutritional value. However, recent studies reported that the gastrointestinal tract (GIT) microbiome plays a major role in the health, physiology, and production traits of ruminants. The rumen is an important digestive organ in ruminants and home to one of the most complex microbial communities, which has long attracted the interest of microbiologists. This organ is rich in bacteria, fungi, and ciliates that ferment forage grass to form volatile fatty acids (VFAs) and microbial proteins (MPs) that provide nutrients for the growth, development, and production of ruminants. The main members of the rumen microbiota (RM) are now well understood. Bacteria account for most species and are geographically widespread in many ruminants and individual animals (1). Ciliate protozoa, which account for up to half of the biomass, are composed of species unique to the rumen (2). The number of anaerobic fungi is relatively small but seems to play an important role in digesting the cell walls of plants that are difficult to break down (3). Archaea are major contributors to methane emissions (4). The RM enhances fiber digestibility, decreases methane emissions, improves the efficiency of nitrogen usage, and also helps explain differences in nutrient digestibility or feed efficiency among animals fed with the same diet. Physiologists and nutritionists have described the rumen's key role in digesting fiber feed and providing nutrition to host animals (5). The intestinal microbiota of beef cattle is also a complex microecosystem. It plays important roles in host material metabolism, immune regulation, biology barriers, and host defense. The number of bacteria in the hindgut system is similar to that in the rumen, and the dominant bacteria in several RM also appear in the normal microbiota of the ruminant large intestine. Characterizing, quantifying, and understanding the role of the RM therefore have significant scientific, economic, and environmental significance. Recent investigations using omics-based approaches reported that RM differences in cattle are associated with production efficacy and health traits. Most RM studies have focused on the microecosystem (6–8). Some have shown that season, animal species, age, diet structure, and other factors can all affect RM (9, 10). However, there are fewer studies on the effect of antibiotics on RM.

16S rRNA sequencing is a quick and easy way to explore the relationship between RM characteristics and animal health (11, 12). It is a validated, rapid, cost-effective approach for analyzing microbial communities and their relevance to environmental factors (13, 14). This technology has been successfully applied to analyze complex bacterial ecosystems in the gut (15).

The discovery and subsequent widespread use of antibiotics controlled infection, saving countless lives, and it played an important role in the prevention and treatment of animal infectious diseases. However, the harm caused by abuse and overuse has attracted wide concern. An imbalance between RM and intestinal microbiota is one of the main adverse reactions, with physiological bacteria greatly reduced and pathogenic bacteria multiplying. Studies have shown that dysbacteriosis (changes in bacterial composition) can lead to the development of digestive, endocrine, psychiatric, systemic, autoimmune, and some infectious diseases (16–20). Ruminants have been recognized as a potential reservoir of antibiotic-resistance genes (21). In addition, including antimicrobials in ruminant diets can select for resistant organisms, potentially modifying the autochthonous RM (22). Doxycycline is a member of the tetracycline class with improved stability and pharmacological efficacy compared to traditional tetracycline (23). This highly effective antibacterial drug has a wide range of applications, good bioavailability, and a few serious adverse events (24). It is mainly used to treat respiratory, urinary, and biliary tract infections caused by sensitive bacteria. Doxycycline is commonly employed in dairy farming and has been used in human and veterinary medicine to fight bacterial infections and promote the growth of food-producing animals, improving feed efficiency and animal performance (25).

Some farmers in China inappropriately give antibiotics to ruminants by oral administration, which can lead to adverse events such as anorexia, belching, regurgitation, severe diarrhea, and even death. This study was conducted to explore the effects of oral antibiotics on the RM. It was designed to assess the distribution and richness of bacterial microbiota in the rumen of beef cattle before and after taking doxycycline. The results show significant RM differences in beef cattle depending on doxycycline administration. Oral doxycycline may alter the RM composition, and bacterial richness may influence corresponding changes that could provide a theoretical basis for the rational and correct use of antibiotics in adult ruminants.

## Methods

### Ethics Statement

All the cows used in this study were treated according to relevant national and international guidelines, and all efforts were made to minimize suffering. The study protocol was approved by the Animal Ethics Committee of Shandong Vocational Animal Science and Veterinary College. No endangered or protected species were involved.

### Animals and Sample Collection

Six healthy, 20-month-old, male Simmental cattle were randomly selected from a beef cattle farm in Shandong province. Animals were kept according to standard beef cattle management methods and fed under standard livestock management practices. The diet feed formulations are shown in Table 1. Three heads were in the experimental group, and three heads served as the control group. The experimental group was fed with doxycycline 20 mg/kg dissolved in 500 ml of 0.9% sodium chloride solution every morning for 6 days. The control group was given 0.9% sodium chloride solution daily. Data show that the feed stays in the rumen for 20–48 h, and the entire digestive process is 40–70 h. After continuous ingestion for 6 days and 2 h after feeding, the rumen contents had undergone at least two cycles. Samples were collected on the seventh day to better assess the effect of doxycycline on multi-rumen microorganisms. On the seventh day, 50-ml rumen fluid samples were collected by inserting a gastric catheter orally after feeding 2 h; they were transported to the laboratory on ice within 2 h and stored at −80°C.

**Table 1:** Beef cattle diet feed formulations.

**Raw material**	**Ratio (%)**
Corn	25.6
Soybean meal	7.15
Bran	6.25
Palm meal	8.25
Corn husk	5.5
Vinasse	5.5
Hay meal	8.7
Silage from whole plants	30.5
Premix	1.25
Salt	0.6
Calcium carbonate	0.7
Total	100
**Dietary nutrient content**	
Dry matter %	70.26
RND/kg	6.32
Crude protein %	12.27
Calcium %	0.67
Phosphorus %	0.34

*RND (beef cattle energy unit) is the calculated value, other components are measured values*.

### DNA Extraction, Amplification, and Sequencing

Genomic DNA was extracted with a TIANamp Genomic DNA Kit (TIANGEN Bio-Tek Co. Ltd., Beijing, China) according to the manufacturer's instructions, and each sample extract was purified with a GeneJET^TM^ Gel Extraction Kit (Thermo Fisher Scientific, Waltham, MA, USA). Generation sequencing library preparation and Illumina MiSeq sequencing were subsequently conducted at Novogene, Inc. (Beijing, China). The 16S rRNA genes of distinct regions (16S V4/16S V3/16S V3-V4/16S V4-V5) were amplified using a specific primer (16S V4: 515F-806R) with the barcode. All polymerase chain reaction (PCR) experiments were performed in 30-μl volumes: 15 μl of Phusion® High-Fidelity PCR Master Mix (New England Biolabs, Ipswich, MA, USA), 0.2 μM of forward and reverse primers, and 10-ng template DNA. Initial denaturation was performed for 1 min at 98°C followed by 30 denaturation cycles at 98°C for 10 s, annealing at 50°C for 30 s, and elongation at 72°C for 30 s. Then samples were held at 72°C for 5 min. In addition to the 16S target-specific sequence, we generated sequencing libraries with Ion Plus Fragment Library Kit 48 rxns (Thermo Fisher Scientific) following the manufacturer's protocol. Library quality was assessed with a Qubit@ 2.0 Fluorometer (Thermo Fisher Scientific). The library was sequenced on an Ion S5 TM XL platform that generated 400-/600-bp single-end reads. The V3, V4, and V5 sequences were processed, spliced, and analyzed by Novogene, Inc.

### Bioinformatics and Statistical Analysis

Single-end reads were assigned based on their unique barcode and truncated by removing the barcode and primer sequence. Quality filtering on raw reads was done under specific filtering conditions to generate high-quality clean reads according to the Cutadapt (26) (V1.9.1, http://cutadapt.readthedocs.io/en/stable/) quality-controlled process. The sample data were separated from the reads obtained according to Barcode, and the Barcode and primer sequences were cut to obtain the original data (raw reads). The reads obtained with the above process still contained chimera sequences. The reads sequence was compared with the species annotation database (https://github.com/torognes/vsearch/) (27) to detect the chimera sequence, which was then removed to obtain the final valid data (clean reads) (28). These were compared with the Silva reference database (https://www.arb-silva.de/) (29) using the UCHIME algorithm (http://www.drive5.com/usearch/manual/uchime_algo.html) (30) to detect chimeric sequences, which were removed (31) to produce clean reads. High-quality sequences were binned into operational taxonomic units (OTUs) with Uparse software (Uparse v7.0.1001, http://drive5.com/uparse/) (32) at a 97% sequence identity threshold. The SSUrRNA of the Silva132 database (https://www.arb-silva.de/) (29, 32) was used based on the Mothur algorithm to annotate taxonomic information for each representative sequence. To study phylogenetic relationships of different OTUs and identify dominant species in samples (groups), we conducted multiple sequence alignment with MUSCLE software (Version 3.8.31, http://www.drive5.com/muscle/) (33). Finally, the data of each sample were homogenized, and those with the least amount of data in the sample were homogenized using a process provided in a script from Novogene, Inc. (there is no specific software). The homogenization process is necessary due to the inconsistency of sequencing depth and the number of sequences between samples. To minimize experimental and human statistical error, we need to set the sequence number of each sample at the same depth level, especially for the comparative analysis between samples. The homogenization method is to set a threshold value (sample with the lowest sequence number), then randomly selected sequence bars set by the threshold value are chosen from the sample for further analysis.

The total number of sequences used to compare each sample is the same, so the relative abundances of species in each sample can be compared. For example, in the following table in the text, the total number of sequences Total_tag minus Uniq_tag is the final number of sequences used for species annotation (Tax_tag+Unclassified_tag). Among them, sampled B1 had the lowest number of sequences at 40,518.

**Table:** 

*#OTU_num*	*qlqlwy1*	*qlqlwy2*	*qlqlwy3*	*qlhlwy1*	*qlhlwy2*	*qlhlwy3*
*#Total_tag*	*71551*	*80159*	*62353*	*70003*	*65758*	*66571*
*#Uniq_tag*	*28559*	*39641*	*21461*	*18986*	*16687*	*20805*
*#Tax_tag*	*42992*	*40518*	*40892*	*51017*	*49071*	*45766*
*#Unclassified_tag*	*0*	*0*	*0*	*0*	*0*	*0*
*#Tax_tag + #Unclassified_tag*	*42992*	*40518*	*40892*	*51017*	*49071*	*45766*

Subsequent analyses of alpha and beta diversity were performed based on these normalized output data. Alpha diversity analysis included the Shannon index, the abundance-based coverage estimator (ACE), and Chao1. Good's coverage index is obtained by adding the number of OTUs with only one sequence and the total number of sequences appearing in the sample in the calculation, so it relatively reflects the sequencing depth of the sample. Beta diversity included both the weighted and unweighted UniFrac values as calculated with QIIME software (Version 1.7.0). Principal component analysis (PCA) was applied to reduce the dimensions of the OTU counts original variables using the FactoMineR and ggplot2 packages in R software (Version 2.15.3). The difference matrixes of OTU abundance of both groups of samples were visualized by principal coordinate analysis (PCoA) to identify principal coordinates and visualize complex, multidimensional data. The distance matrixes of weighted or unweighted UniFrac values were transformed to a new set of orthogonal axes, by which the maximum variation factor is demonstrated by first principal coordinate, the second maximum factor by the second principal coordinate, etc. The PCoA results were displayed with the WGCNA, stat, and ggplot2 packages in R software (Version 2.15.3). To assess similarity between two groups of samples, a tree was constructed by clustering. In environmental biology, UPGMA (Unweighted Pair-group Method with Arithmetic Mean) is a commonly used cluster analysis method that was first used to solve the classification problem. The basic concept of UPGMA is as follows. Identify the two samples with the smallest distance and form a new node (new sample) with a branch point located at half the distance between the two samples; then calculate the new average distance between the “sample” and other samples, and then find the smallest two samples for clustering. This process is repeated until all the samples come together in a complete clustering tree. UPGMA cluster analysis is performed using weighted and unweighted UniFrac distance matrixes, and the clustering results are integrated with the relative abundance of species at the gate level for each sample. UPGMA clustering was carried out as a form of hierarchical clustering to interpret the distance matrix using average linkage in QIIME software (Version 1.7.0). ANOSIM is the analysis of similarity; this nonparametric test is used to examine whether the difference between groups is significantly greater than the difference within groups to determine if there is clear clustering from the analysis of the distance matrix (34).

The size of the intragroup differences can be used to determine whether the grouping is meaningful and to test inter- and intragroup differences between two groups or among more groups. ANOSIM uses the R vegan package (ANOSIM function) based on the Bray–Curtis distance value. The ANOSIM results showed that the R-value was between −1 and 1.

$$\begin{array}{l} R=\frac{r_{b}-r_{w}}{\frac{1}{4}\left[n\left(n-1\right)\right]} \end{array}$$

where *r*_*b*_ is the mean rank of between group dissimilarities, *r*_*w*_ is the mean rank of within group dissimilarities, and *n* is the number of samples.

An *R* > 0 indicates that the similarity within groups is lower than the similarity between groups. An *R* < 0 indicates that the similarity between groups is lower than that within groups. Significance testing was performed to identify differences between groups, which were considered significant at *P* < 0.05. Data are given as mean ± standard deviation (SD) and were analyzed with SPSS 17.0 software (SPSS Inc., Chicago, IL, USA).

## Results and Discussion

### Microbial Diversity Index Analysis of Rumens With or Without Doxycycline

**Figure 1:**
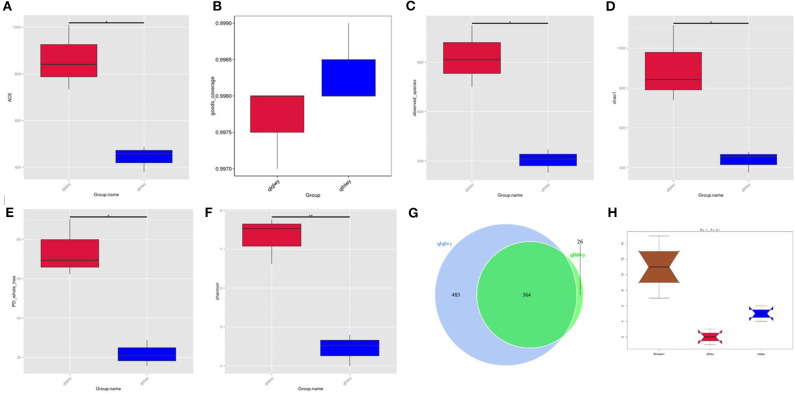
Collation results of DNA sequence data and microbial diversity index analyses. **(A)** ACE index, **(B)** Good's coverage index, **(C)** observed species index, **(D)** PD whole tree, **(E)** Chao1 index, **(F)** Shannon index, **(G)** Venn diagram. The numbers represent the unique or common OTUs of each group. **(H)** ANOSIM analysis. “Between” represents the difference between groups; an R-value closer to 1 indicates greater difference. “qlqlwy” and “qlhlwy” represent the two groups.

Rumen contents were collected for high-throughput sequencing to assess bacterial community composition in rumens of beef cattle with or without oral doxycycline. To study the species composition of each sample, we included OTUs with 97% identity on the valid labels of all samples, clustered the OTUs, and then annotated the OTU sequences. The 97% identity value refers to the comparison between the read and reference sequences. An OTU is defined as a read with 97% nucleotide sequence identity. Based on 97% species similarity, 1,047 and 590 OTUs were obtained from samples from the qlqlwy and qlhlwy groups, respectively. Among all samples, there were 1,073 OTUs, of which 564 in both groups were defined as core OTUs (52.56% of all OTUs, Figure 1G). The representative sequence was selected by removing the effective tags, and the singletons were arranged according to the abundance and clustered according to 97% similarity. The representative sequence is the one with the highest frequency. In addition, 26 OTUs were uniquely identified only in group qlhlwy.

Bacterial diversity and richness (alpha diversity measurements) were assessed with the Shannon index, Chao1, ACE, and Good's coverage. Good's coverage for each sample was >99.75% (Figure 1B), demonstrating that the 16SrDNA sequences in these samples represent most bacteria present. The highest microbial richness was in the rumens without doxycycline; the average Chao1 index was 899.17 (Figure 1D), and the average ACE index was 862.011 (Figure 1A). The richness of rumens from cattle treated with doxycycline was lower than in those without doxycycline, and the average Chao1 and ACE indexes were 436.38 and 441.65 (Figures 1A,D), respectively. Similarly, the Shannon indexes in rumen samples from untreated and treated cattle were 7.312 and 4.437, respectively (Figure 1F). Moreover, the observed species and phylogenetic diversity (PD) whole tree of rumens were more abundant in the untreated group (Figures 1C,E). Consistently, the rumen of doxycycline-treated cattle had lower Simpson diversity index values. Furthermore, ANOSIM results showed that between-group differences were greater than those within groups (*R* = 1, *P* = 0.1; Figure 1H). For community richness comparisons, both ACE and Chao1 showed that the rumens of untreated cattle contained significantly more observed and estimated OTUs than doxycycline-treated cows. This result demonstrates that doxycycline reduces bacterial diversity and abundance in the rumen of beef cattle.

### Beta-Diversity Analysis of the Microbial Communities of Rumens With or Without Doxycycline

**Figure 2:**
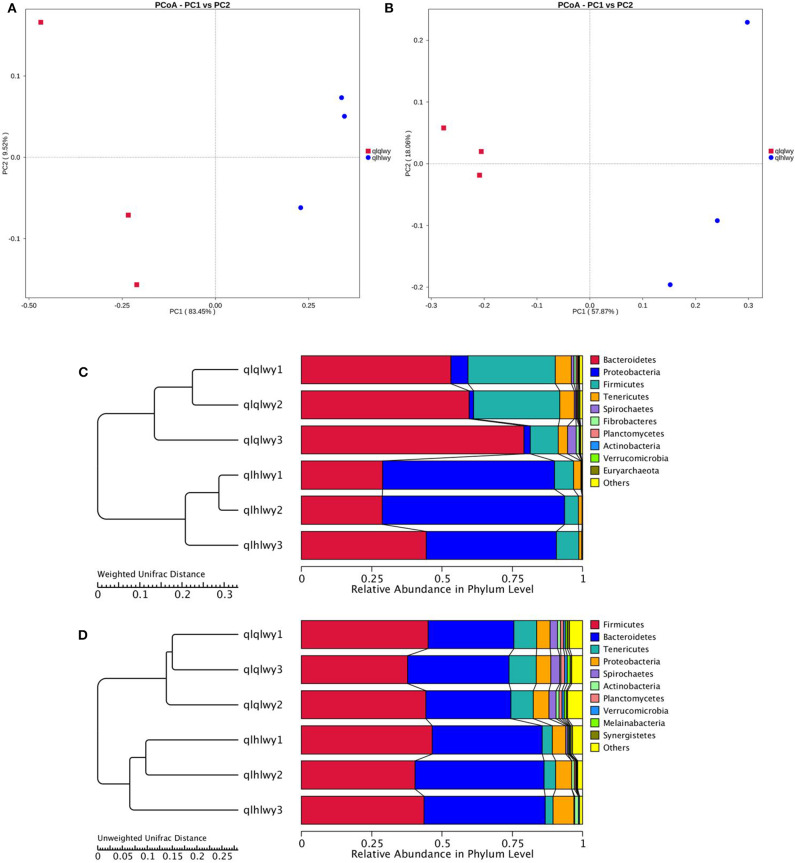
Differences in bacterial community structures and relationships among all samples. **(A,B)** PCoA of bacterial community structures of the RM in the three sample groups. Each blue point represents a sample. The distance between two points represents the difference of RM. PCoA shows distinct bacterial communities between different samples. **(C)** The UPGMA tree analysis of samples in evolution in weighted unifrac distance. **(D)** The UPGMA tree analysis of samples in evolution in unweighted unifrac distance.

The relationships between the community structures of beef cattle RM were examined using PCoA. The UniFrac distance matrix revealed clear differences among all individual samples and groups. The microbiota in each group were clustered. Figures 2A,B depict the weighted and unweighted UniFrac distances of PCoA analyses, respectively, and show that the RM of doxycycline-treated cattle were distinct from untreated samples. The relationships among community structures as revealed by PCoA were further examined by assessing between-group weighted UniFrac distances and the UPGMA tree. Consistent with the PCoA plot, the UPGMA tree showed significantly different microbial community structures between groups qlqlwy and qlhlwy for weighted UniFrac distance (Figure 2C) but not unweighted Unifrac distance (Figure 2D).

**Figure 3:**
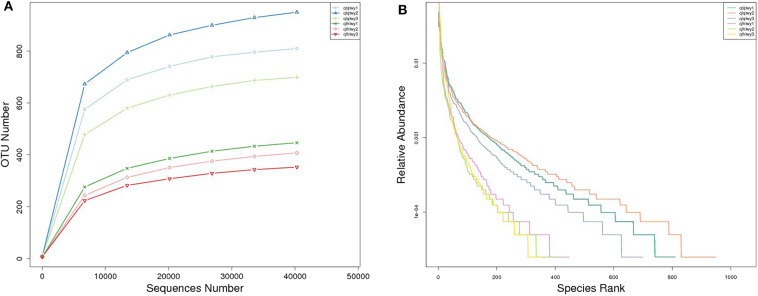
Sample feasibility analysis. Each curve represents a sample. **(A)** Rarefaction curves depicting the effect of sequences on the number of identified OTUs in the rank abundance curve. **(B)** The abscissa indicates the OTU (species) abundance order, and the ordinate corresponds to the relative abundance ratio of OTU (species).

A rank abundance curve was generated to further demonstrate species abundance and evenness. In group qlqlwy RM samples, the OTU ranks were ~800 more than those of group qlhlwy, which were close to 400 (Figure 3B), indicating less abundant species compositions in group qlhlwy samples. All curves were relatively flat, indicating relative uniform species compositions for all samples (Figure 3A). These curves tend to be flat when the number of effective sequences reaches 30,000. The number of valid sequences of each sample was >40,000, which indicated sufficient sequencing data.

### Bacterial Community Composition at Different Taxonomical Levels

**Figure 4:**
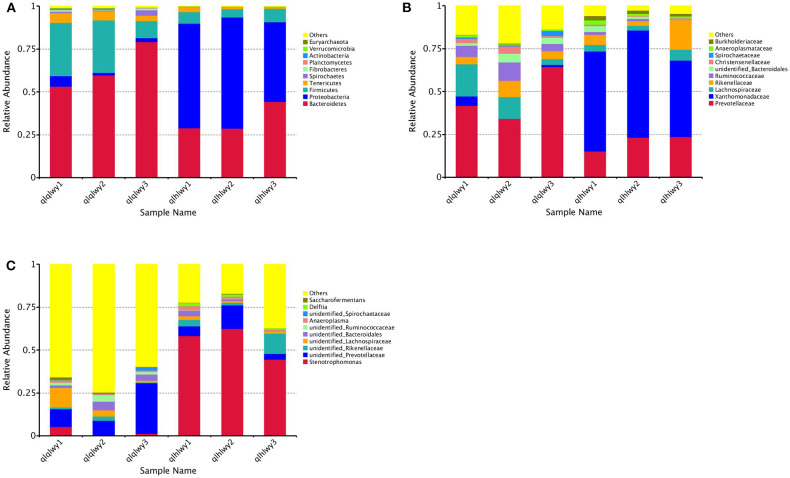
Microbial composition of different samples. Each bar represents the average relative abundance of each bacterial taxon within a group. The top 11 abundant taxa are shown. **(A)** Taxa assignments at Phylum level. **(B)** Taxa assignments at Family level. **(C)** Taxa assignments at Genus level.

We next analyzed rumen bacterial community composition and structure by taxonomical level. According to the phylum assignment result, Bacteroidetes was the predominant phylum in group qlqlwy samples, whereas Proteobacteria was the governing phylum in group qlhlwy. Firmicutes and Bacteroidetes were the secondary phyla for groups qlqlwy and qlhlwy, respectively (Figure 4A). Bacterial abundance was also analyzed for family (Figure 4B) and genus (Figure 4C). On the family level, there were significant between-group differences. The top three predominant populations in group qlqlwy were Prevotellaceae, Lachnospiraceae, and Ruminococcaceae, compared to Xanthomonadaceae, Prevotellaceae, and Rikenellaceae for group qlhlwy (Figure 4B). At the genus level, there were also significant differences among three samples from two groups. The top predominant population in group qlqlwy was unidentified_Prevotellaceae. However, in group qlhlwy, the most common genus was Stenotrophomonas (Figure 4C). The most important factor is that there were obvious changes between unidentified_Prevotellaceae and Stenotrophomonas with treatment. The proportion of unidentified_Prevotellaceae gradually decreased with doxycycline, while Stenotrophomonas increased (Figure 4C). This finding demonstrates that doxycycline treatment clearly affects the bacterial community composition of the rumen in beef cattle.

### Differences in Bacterial Communities Between Rumens With or Without Doxycycline

**Figure 5:**
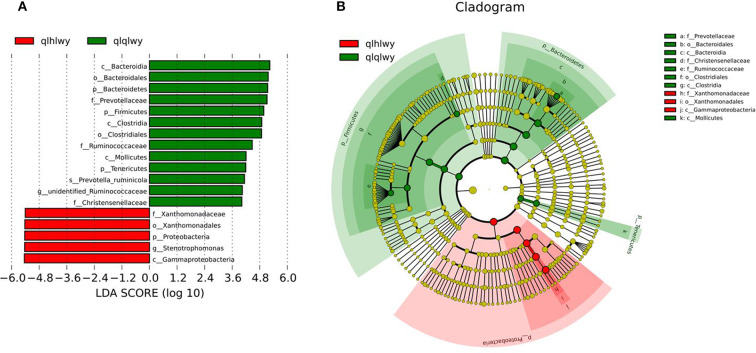
Bacterial taxa significantly differentiated rumens samples of cows with or without doxycycline identified by LEfSe using the default parameters. **(A)** Histogram of LDA scores computed for bacterial taxa differentially abundant between groups. **(B)** Bacterial taxa that were differentially abundant visualized using a cladogram.

Linear discriminant analysis (LDA) effect size (LEfSe) was applied to the top 18 taxa (average relative abundance >0.00001) to determine which were significantly different between groups (Figure 5A). The bacterial taxa of rumens differentially represented the groups qlqlwy and qlhlwy. Eight and three bacterial taxa were significantly more abundant in qlqlwy and qlhlwy, respectively (Figure 5B). The RM in group qlqlwy was significantly more diverse in both species and relative abundance. Bacteroides was the most abundant taxa in group qlqlwy, whereas Xanthomonadaceae was the most common in group qlhlwy. These are consistent with the bacterial community compositions of the rumen samples described above.

### Correlation of RM in Beef Cattle With or Without Doxycycline

**Figure 6:**
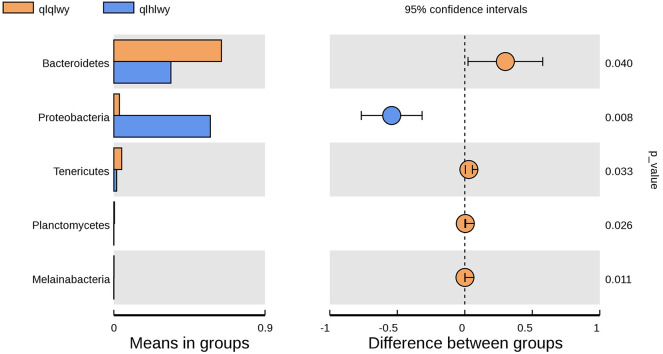
Differences in bacterial abundance between the groups qlqlwy and qlhlwy. Left, abundance ratios of different strains in two samples. Middle, difference in bacterial abundance within the 95% confidence interval. Right, *P*-values of significance testing.

There were significant differences in the diversity and abundance of intestinal microbiota following doxycycline treatment. To investigate the effect of doxycycline on RM, we performed a statistical analysis of metagenomic profiles (STAMP, Figure 6). There were no significant differences in other RM between the two groups except for Bacteroidetes (*P* = 0.04) and Proteobacteria (*P* = 0.008). Bacteroidetes was previously determined to have a high abundance in beef cattle without doxycycline, indicating that these are necessary for normal animals (Figure 4A). In addition, Proteobacteria had high abundance in doxycycline-treated animals, but the proportion of Bacteroidetes decreased, suggesting that doxycycline can promote Proteobacteria growth, and inhibit Bacteroidetes survival. Tenericutes, Planctomycetes, and Melainabacteria in group qlhwly also showed decreasing trends. Despite their low abundance, these changes should not be ignored.

The gastrointestinal microbiota is called the “second genome” and plays an important role in animal growth and health, especially in ruminants. Previous studies have shown that gastrointestinal microbes can influence body weight and digestion and decrease the risks of infection and autoimmune diseases. The intestinal microbiota has been linked to several conditions including diabetes and inflammatory bowel disease (35, 36). It was also reported that there was a significant difference in the bacterial composition of BALBc mice receiving comparable immune programs in specific pathogen-free units of different centers, which supported the role of intestinal microbiota in regulating the induction response (37). Over time, the RM has been investigated in sheep, cattle, and other ruminants. Mammalian gastrointestinal studies have examined the effects of the microbiota on metabolism, physiology, and immunology (38, 39), but there are a few reports of RM differences in cattle following antibiotic administration. Here, we analyzed bacterial diversity and abundance in the contents of cattle RM after doxycycline treatment. The results showed that the abundance and diversity of bacteria in the RM of cattle on doxycycline were lower than in untreated cattle.

The RM of dairy beef cattle is not present at birth, but as the animals are in constant contact with the external environment, it gradually colonizes, survives, and reproduces after adapting to the environment. Cattle RM reportedly increase in diversity and tend to be composed of mature bacteria as animals age (40).

The present results demonstrate significant differences in the RM after doxycycline treatment. Prior to antibiotic administration, Bacteroidetes and Firmicutes were the most common phyla in the rumen samples of group qlqlwy, which is consistent with a previous report (40). After doxycycline administration, Proteobacteria became the most abundant phylum in group qlhlwy. The proportion of Bacteroides was small. The dominant bacteria in the RM are mainly composed of Bacteroides, Proteobacteria, and Firmicutes, but their proportions vary greatly.

Gene functions in the intestinal microbiota of healthy humans may be more diverse than previously hypothesized, and the main axis of taxonomic variation in the microbiome may not capture the largest functional variation (41). Bacteroides are beneficial intestinal microbiota because they break down polysaccharides and improve nutrient utilization (42), degrade carbohydrates and proteins, and promote the development of the gastrointestinal immune system (43). All these events strengthen the host's immune system (44). In ruminants, Firmicutes is involved in degrading fiber and cellulose (45) and maintaining an appropriate intestinal micro-ecological balance (46).

Since intestinal microbiota imbalance is usually caused by a continuously increased abundance of Proteobacteria, the physiological human intestinal microbiota contains only a small proportion of that phylum. Increased prevalence of Proteus could be a useful diagnostic marker for dysbiosis and disease risk (47). In support of the proposed relationship between metabolic disorder and Proteobacteria expansion, a mono-association study of germ-free mice revealed an obesogenic potential of Proteobacteria (48). In fact, a growing body of evidence suggests that an abundance of Proteobacteria members may be a pathogenetic feature. This feature has known associations with metabolic disorders and inflammatory bowel diseases, but it may also play a role in lung diseases including asthma and chronic obstructive pulmonary disease. All of these conditions have varying degrees of inflammation.

Many studies have confirmed that the use of antibiotics and some drugs can alter the RM of dairy beef cattle. Li et al. (49) fed pasteurized antibiotic milk, antibiotic milk, or fresh milk to 2-, 3-, and 6-month-old calves. Antibiotic milk gradually increased Firmicutes abundance, while Bacteroides gradually decreased. There were also significant proteobacteria differences in each group. Shen et al. (50) demonstrated that monensin and nisin both reduced the numbers of bacteria, fungi, and methanogens. It is undeniable that antibiotics have strong effects against dangerous pathogens, but they also damage the beneficial bacteria colonized in the rumen and change the steady state of rumen microorganisms, especially when taken orally. Therefore, it is necessary to judiciously use antibiotics in dairy cow production. When there is no choice, injection is preferred over oral administration to minimize damage to the RM.

The number of animals selected for this study was limited due to the research cost. Our results should therefore be confirmed in a larger sample of animals.

## Data Availability Statement

The datasets generated for this study can be found in NCBI SRA, NCBI Accession Nos. PRJNA624243 and PRJNA624271.

## Ethics Statement

The animal study was reviewed and approved by Shandong Vocational Animal Science and Veterinary College. Written informed consent was obtained from the owners for the participation of their animals in this study.

## Author Contributions

All authors designed the subject, collected the sample, analysed, and wrote the manuscript.

## Conflict of Interest

The authors declare that the research was conducted in the absence of any commercial or financial relationships that could be construed as a potential conflict of interest.
